# Supplementary material for: Real-Time Feeding Behavior Monitoring by Electrical Penetration Graph Rapidly Reveals Host Plant Susceptibility to Crapemyrtle Bark Scale (Hemiptera: Eriococcidae)
Source: Insects. 2022 May 25;13(6):495. doi: 10.3390/insects13060495 (PMC9224517; doi:10.3390/insects13060495)
Supplement: Supplementary file 1 [file insects-13-00495-s001.zip › insects-1715817-supplementary.pdf]

Supplementary Materials

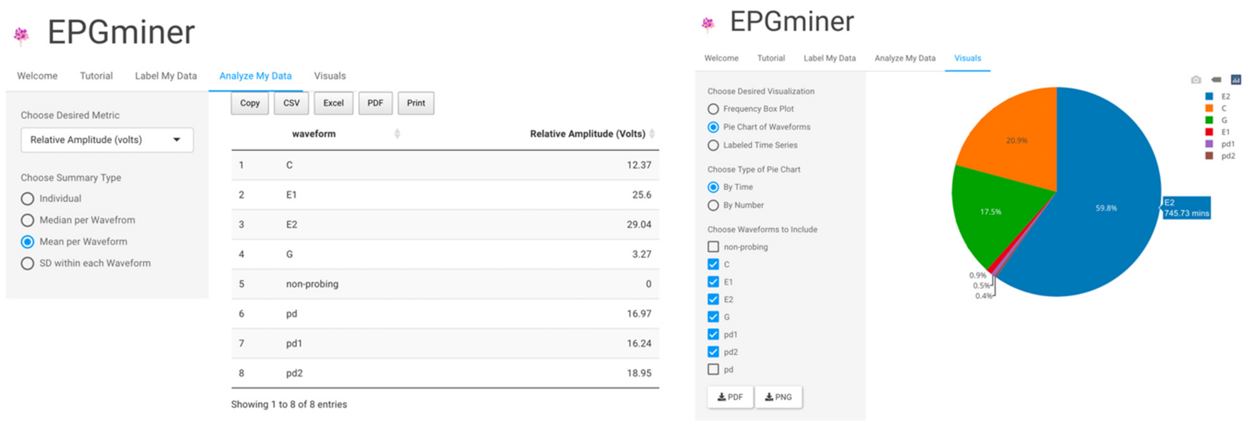

Figure S1. EPGminer application released on Zenodo and GitHub assists in semi-automatical calculation of feeding characteristics and visualization of EPG analysis results for feeding behavior study of *Acanthococcus lagerstroemiae*. EPGminer has the website version https://epgdata.shinyapps.io/epgminer_app/ and the software version. R package code is available in Zenodo: https://doi.org/10.5281/zenodo.6299917. https://github.com/LylChun/epgminer/tree/master/inst/epgminer_app/rsconnect/shinyapps.io/epgdata. Detailed instructions on EPGminer utilization (step-by-step demo included) in the calculation of frequency and relative amplitude for each waveform are provided in GitHub https://lylchun.github.io/EPGminer/index.html.

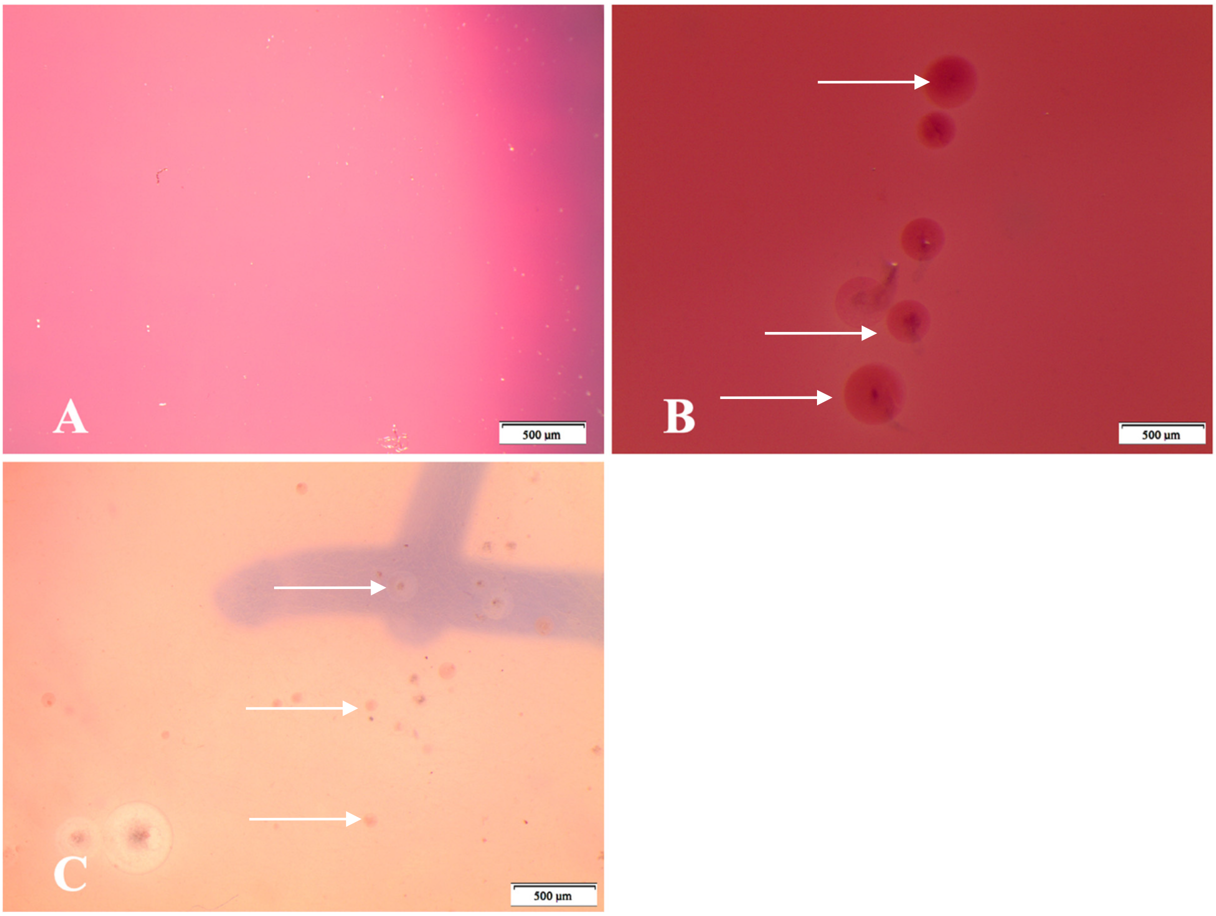

Figure S2. Overview of pectinesterase detection in agarose gel plates. (**A**) No rings or halos were shown when *A. lagerstroemiae* was feeding on the plates with no pectin added. (**B**) Maroon-stained rings and halos (arrows), representing pectinesterase activity, were shown by using ruthenium red around the feeding site of the stylet sheath when *A. lagerstroemiae* was feeding on 0.1% pectin-added gel plates. (**C**) The number and size of rings and halos in 1.0% pectin-added plates were not different from 0.1% pectin plates.
